# Deep Convolutional Neural Networks for Classifying Body Constitution Based on Face Image

**DOI:** 10.1155/2017/9846707

**Published:** 2017-10-18

**Authors:** Er-Yang Huan, Gui-Hua Wen, Shi-Jun Zhang, Dan-Yang Li, Yang Hu, Tian-Yuan Chang, Qing Wang, Bing-Lin Huang

**Affiliations:** ^1^School of Computer Science and Engineering, South China University of Technology, Guangzhou 510006, China; ^2^Department of TCM, The First Affiliated Hospital of Sun Yat-sen University, Guangzhou 510080, China

## Abstract

Body constitution classification is the basis and core content of traditional Chinese medicine constitution research. It is to extract the relevant laws from the complex constitution phenomenon and finally build the constitution classification system. Traditional identification methods have the disadvantages of inefficiency and low accuracy, for instance, questionnaires. This paper proposed a body constitution recognition algorithm based on deep convolutional neural network, which can classify individual constitution types according to face images. The proposed model first uses the convolutional neural network to extract the features of face image and then combines the extracted features with the color features. Finally, the fusion features are input to the Softmax classifier to get the classification result. Different comparison experiments show that the algorithm proposed in this paper can achieve the accuracy of 65.29% about the constitution classification. And its performance was accepted by Chinese medicine practitioners.

## 1. Introduction

Traditional Chinese medicine (TCM) constitution theory originated in the “Yellow Emperor” <黄帝内经>. Through the enrichment and development of constitution physiology, traditional Chinese physicians have gradually constructed a relatively independent discipline system of TCM constitution and clarified the concept of TCM constitution in the 1970s. Traditional Chinese Medicine constitution physiology is based on the theory of traditional Chinese medicine, through the study of various human constitutional characteristics and constitutional types of physiological and pathological characteristics, then analyzing the disease response state, the nature of the disease and development trends, and finally guiding disease prevention, treatment, and health rehabilitation. Body constitution is an objective life phenomenon, which shows the morphological structure, physiological function, psychological state, and other aspects of the comprehensive, relatively stable characteristics in the individual life process. This character determines the body's susceptibility to certain pathogenic factors and the tendency of its type of disease. The difference in constitution is the result of congenital factors and a variety of acquired factors [1, 2].

Constitution classification is the basis and core content of TCM constitution research. It is to extract the relevant laws from the complex constitution phenomenon and finally build the constitution classification system. The commonly used constitution types are determined by the traditional Chinese medicine constitutional questionnaire which is developed by Wang [3] in the mainland and Lin et al. [4–8] in Taiwan. Wang's research of body constitution has been the standard for Chinese medical diagnosis and treatment. He divided the constitution into nine types. Body constitutions are classified as gentleness, Qi-deficiency, Qi-depression, dampness-heat, phlegm-dampness, blood-stasis, special-diathesis, Yang-deficiency, and Yin-deficiency [3]. In 2009, the Chinese Association of Chinese Medicine issued the “nine kinds of constitution measurement table,” which became the standard method of Chinese constitution research [9]. With the current “classification and determination of Chinese medicine constitution” standard in the field of traditional Chinese medicine, practitioners continue to use it, and its shortcomings are gradually emerging [10, 11].

1. It is influenced by subjective factors. At present, the method of constitution classification is mainly through the determination of the constitution measurement table, and it can be said that the basic diagnosis is based on the consultation of Chinese medicine four clinics. There are a large part of the subjective factors, such as the accuracy of the collected data, the respondents' experience, and the degree of understanding of the respondents.
2. The number of questions is too large and takes a long time, so that many respondents lose their patience in the process of filling out the constitution measurement table; then these elements have an impact on constitution classification.
3. Score calculation formula is more complex, so that many people cannot accurately calculate their constitution type.

It is necessary to develop computer technology to standardize and objectify the constitutional diagnosis method in order to solve the above problems. At present, many scholars have applied biology knowledge and machine learning algorithm to TCM diagnosis process [12–16]. Zhao et al. [17] discussed the research of machine learning and TCM diagnosis so as to further study the classification of patients. Inspired by the brain hierarchy, many researchers have been working on multilayer neural networks. Wang and Bai [18] applied the BP neural network to pulse diagnosis to classify the type of constitution and demonstrated the rationality and superiority of this method. Liu et al. [19] argued that deep learning is clearly more in line with human brain and can use high-dimensional abstract features to represent some of the original low-dimensional features. It is a good way to find the relationship between the symptoms and syndromes. This idea is consistent with the diagnosis of traditional Chinese medicine. So they use the deep learning and multilabel learning methods to build one model used to diagnose the chronic gastritis in traditional Chinese medicine. At the same time, deep convolution neural network is mainly used in image recognition and shows good results [20–23]. Hu et al. [24] applied the convolution neural network to the pulse diagnosis. In the case of feature ambiguity, the proposed method is superior to other well-known methods. Li et al. [25] used the convolution neural network to extract the features of the pulse and then classify the body constitution type. The experimental results show that this method can obtain high accuracy.

Therefore, this paper presents the deep convolutional neural networks for classifying body constitution based on face image. The second section will introduce the collected face dataset, the convolution neural network algorithm, and some commonly used pattern recognition algorithms. The evaluation procedure, the obtained experimental results, and discussion are presented in Section 3. Finally, some conclusions are given in Section 4.

## 2. Method

**Figure 1:**
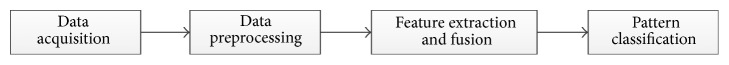
The flow chart of the whole algorithm.

The algorithm proposed in this paper is divided into four main parts: (1) data acquisition, (2) data preprocessing, (3) features extraction and fusion, and (4) pattern classification algorithm. The flow chart of the whole algorithm is shown in Figure 1. First, collect the face image data set and preprocess the picture. Then, the face feature is extracted by convolution neural network, and these features are merged with the face color feature. Finally, the pattern recognition algorithm is used for constitution recognition. The following sections provide a detailed description of the modules contained in the architecture.

### 2.1. Data Acquisition and Preprocessing

There are 5330 face images used in this article. The face dataset is collected by capturing the patient's face picture in the three hospitals of the Chinese medicine outpatient department, respectively. The type of body constitution is judged by a medical professor in each TCM outpatient room. The judgment is based on the standard of classification and determination of constitution in TCM which is developed by Professor Wang [3]. This standard has been listed as China's national standard. Before collecting data, the standard is discussed by nearly ten medical experts. Some agreed with this standard. Some professors were partially in favor of the standard. Some professors have a negative attitude on this standard. We chose three professors who were in favor of this standard. This means that they reached the consensus (agreement of standard) to determine the type of body constitution. Subsequently, they were in different hospitals to judge the patient's body constitution according to the standard. In this way, the impact of experience can be reduced as much as possible. Besides, these professors are well known and their ages are close, and the personal experience is not greatly different. Finally, the body constitution type of the patient in the same hospital is determined by the same medical professor. The entire dataset is determined by three Chinese medicine professors from three different hospitals according to the above-mentioned standard.

On the other hand, it is proved in practice that the reliability and validity of the diagnosis by these Chinese medicine professors are better than those of the questionnaire survey method for the body constitution identification by Wang's questionnaire (CCMQ). Now CCMQ systems have been deployed in many hospitals. The survey shows that the actual utilization rate is not high, and the main reason is from the patient's subjective problem, instead of CCMQ standard itself. The subjective factors of the patients are mainly influenced in three aspects. (1) Patients do not want to really answer the question because of privacy considerations. (2) Patients are not medical experts. They lacked medical knowledge to some questions so that their answers are easily wrong. (3) It costs too much time to answer these questions, so many patients feel impatient and tired, leading to random answers.

Therefore, all face images are taken by the same type of digital device and the patient's physical type is specified by the doctor. The indoor environment is no sunshine, and lighting conditions are normal fluorescent lamps. In the face database, there are 8 kinds of constitution types, that is, gentleness, Qi-deficiency, Qi-depression, dampness-heat, phlegm-dampness, blood-stasis, Yang-deficiency, and Yin-deficiency. The number of each constitutional type is shown in Table 1. In the preprocessing process, firstly, the face detection algorithm is used to detect the acquired picture, and the corresponding bounding box is obtained. Considering both time complexity and precision, this paper uses OpenCV tools to complete the face detection.

### 2.2. Features Extraction

Traditional Chinese medicine (TCM) is based on more than 2,500 years of Chinese medical practice. The diagnostic principle of traditional Chinese medicine is based on information obtained from four diagnostic procedures, namely, diagnosis through observation, diagnosis through auscultation and olfaction, diagnosis through inquiry, and diagnosis through pulse feeling and palpation [26]. The diagnosis through observation is mainly based on the face of the tongue, skin color, and other information to determine [27]. The algorithm proposed in this paper is used to extract the features of face images for body constitutional identification, mainly with color and convolution neural network algorithm for feature representation.

#### 2.2.1. Color Feature

The color is a very important visual feature of the image. Compared with other features, the color feature is not sensitive to the translation, scale, and rotation of the image, and it is very robust and simple to calculate. In this paper, we use the method of color histogram based on HSV color space to extract the color feature. From the psychological perception of people, the HSV space is more intuitive and easier to accept compared with the RGB space [28]. The hue describes the properties of the solid color; the saturation is used to measure the extent to which solid color is diluted with white light; the value indicates the brightness of the color. The conversion relationship between RGB space and HSV space is as follows.

(1) V=max⁡R,G,B,S=V−min⁡R,G,BV,if V≠0,0,otherwise,H=60G−BV−min⁡R,G,B,if V=R,120+60B−RV−min⁡R,G,B,if V=G,240+60R−GV−min⁡R,G,B,if V=B.

#### 2.2.2. CNN Feature

The convolution neural network (CNN) [29] is an effective method of autonomous learning in deep learning. It can reconstruct the high-level semantic features from the original image and improve the training performance by weight sharing. The convoluted neural network consists of alternating layers of convoluted and pooled layers, simulating simple cell and complex cell cascade structures for high-level feature extraction in the visual cortex. The neurons of the convolution layer respond to a portion of the region of the previous layer and extract the higher features of the input. The neurons of the pooled layer are averaged or maximized for a portion of the input of the previous layer, resisting the slight deformation or displacement. The latter layers of the convoluted neural network are typically a number of fully connected layers and a classifier. In recent years, convolution neural networks have been successfully applied to facial expression recognition [30, 31], face recognition [32, 33], human posture estimation [34], age estimation [35, 36], and speech recognition [37, 38].

The convolutional neural network is a feature-based method and applied to physical recognition. It is different from the traditional artificial feature extraction and the high performance classifier design for the feature. Its advantage is that the feature extraction is carried out by layer-by-layer convolution and dimensionality. And then through the multilayer nonlinear mapping, the network can automatically learn to form the identification task for the feature extractor and classifier from the training sample. The method reduces the requirement of the training sample, and the more the network layer is, the more the characteristic of the learning is more global.

**Figure 2:**
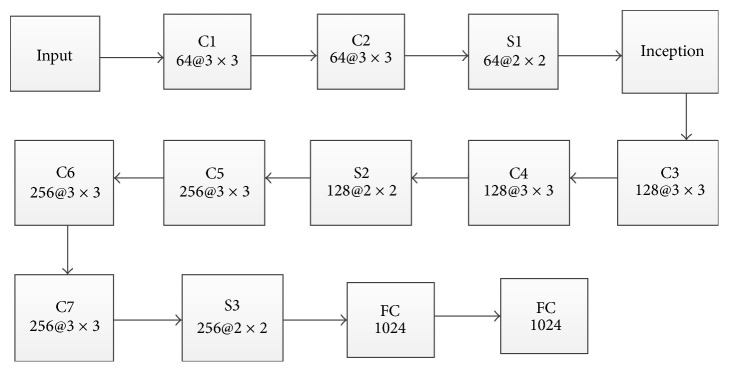
The structure of convolutional neural networks for extracting features.

This paper is inspired by the literature [39] to construct a deep convolution neural network to study facial features. The specific network structure is shown in Figure 2. The network consists of seven convolutional layers, three pooling layers, one inception layer, and two fully connected layers. The size of the input image is 48 × 48, the size of kernel is 3 × 3, and the number of feature maps is different. The pooling layer selects MaxPooling and the pooling area size is 2 × 2. The dropout [40] and MaxPooling operations are designed to further reduce network parameters and prevent network overfitting. The inception layer not only increases the width of the network, but also increases the adaptability of the network to the scale by increasing the convolution operation of 1 × 1, 3 × 3, and 5 × 5 and the pooling operation of 3 × 3. The last two layers are the full connected layers, and the size is 1024. The face feature used in this paper is the last full connected layer.

### 2.3. Classification Algorithms

Pattern classification can be carried out to classify the faces into different types by the features, such as color features, texture features, and features extracted by the CNN model. There are many algorithms in pattern classification, such as Naive Bayes classifier [41], support vector machine classifier [42], Random Forest classifier [43], KNN classifier [44], Decision Tree classifier [45], Logistic Regression classifier [46], and Gradient Descent Boosting classifier [47]. By extracting the features in Section 2.2 and then entering the different classifiers, different classification results are obtained. In Section 3, we will compare the effect of these classifiers in detail.

## 3. Experiment

In this section, we conducted a series of experiments to measure the effectiveness of the body constitution recognition algorithm. The details of these experiments are described below.

### 3.1. Experiment Settings

The tools used in this experiment are based on Keras and Scikit-learn [48, 49]. The GPU is NVIDIA GTX Titan X, and its memory size is 12 GB. The operating system is Ubuntu 14.04. The face dataset used in this paper has 5330 images, of which 90% of the image is a training set and the remaining 10% of the image is a test set. In the process of training, the value of dropout is set to 0.5 after pool layer and the full connection layer in order to prevent overfitting. The whole network is trained by random gradient method. The learning rate is 0.01, the momentum is set to 0.9, and the batch size is set to 50.

### 3.2. Experiment Results

In this paper, we first extract the color and texture features and the features extracted by the convolution neural network and then compare the classification effect of the feature extraction method under a classification algorithm. Among them, the support vector machine in the kernel function is to select RBF, and the value of *K* is set to 5 in the KNN method. Under the premise of the same classifier, the classification effect of fusion color and texture feature is better than that based on single color feature, and the classification effect based on convolution neural network is better than that based on color and texture feature fusion in Table 2. At the same time, under the same feature extraction method, the classification effect of different classifiers is compared. Based on the premise of a single color feature, KNN classification accuracy is the best. Under the premise of color and texture feature fusion, random forest classification is the best. Based on the feature extraction method of convolution neural network, the classification effect of Softmax is the best. The confusion matrix of random forest classification based on color texture feature fusion is in Table 3. The confusion matrix of Softmax classification based on convolutional neural network is in Table 4. As can be seen from Table 4, the algorithm is not very good for the classification of gentleness, by the great impact of Qi-deficiency.

The color is a basis for judgment in the diagnosis through observation. Therefore, this paper proposes a method of combining the features of the convolution neural network and the color features, and the classification results are shown in Table 5. As can be seen from Table 5, the Softmax has the best classification effect under the premise of the feature of convolution neural network and color feature fusion. The confusion matrix for this method is shown in Table 6.

**Figure 3:**
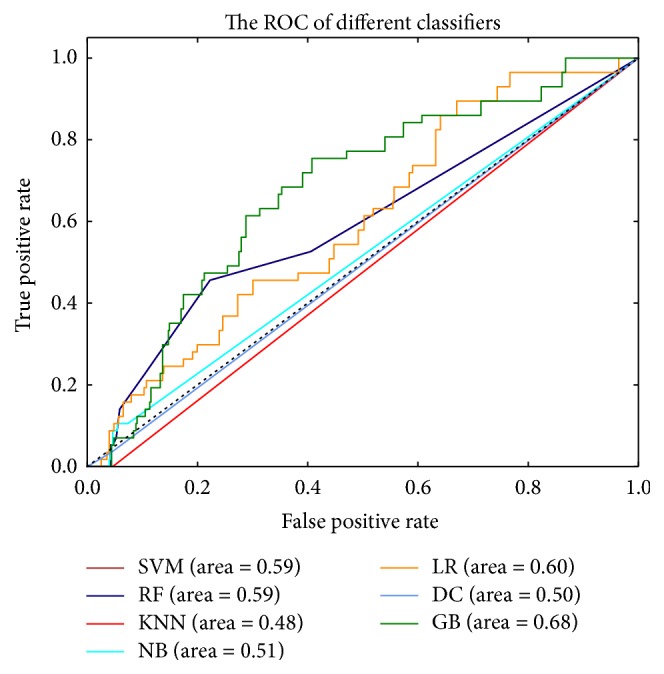
The ROC curve of different classifiers based on the feature of convolution neural network and color feature fusion. The dotted black line is the baseline in ROC curve. It indicates that the true positive rate (TPR) is equal to the false positive rate (FPR).

**Figure 4:**
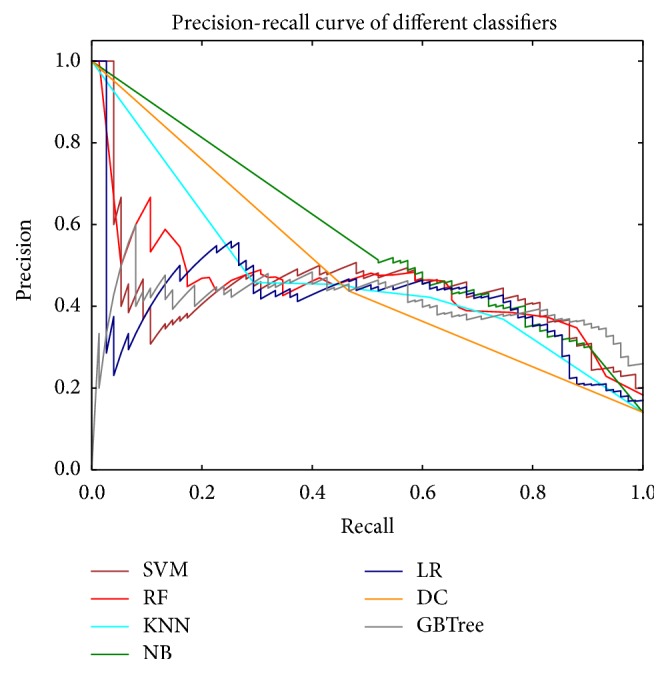
The precision-recall curve of different classifiers based on the feature of convolution neural network and color feature fusion.

**Figure 5:**
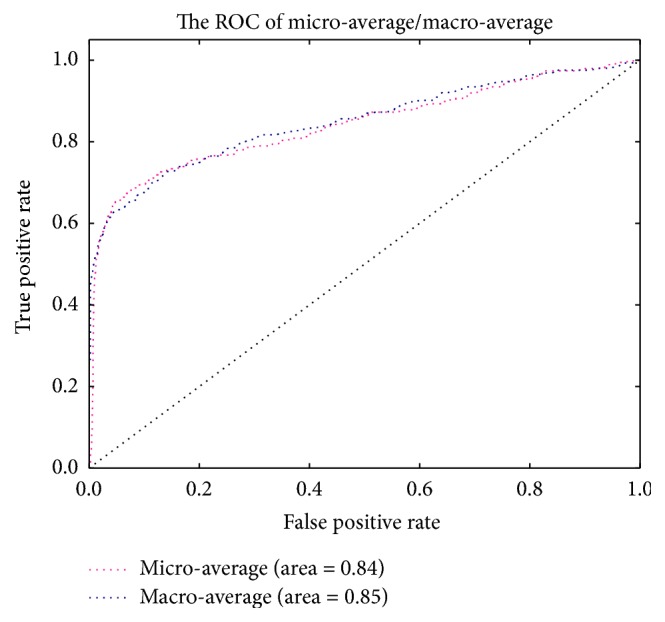
The micro-average and macro-average ROC curve in the Softmax based on the convolution neural network and the color feature fusion. The dotted black line is the baseline in ROC curve. It indicates that the true positive rate (TPR) is equal to the false positive rate (FPR).

**Figure 6:**
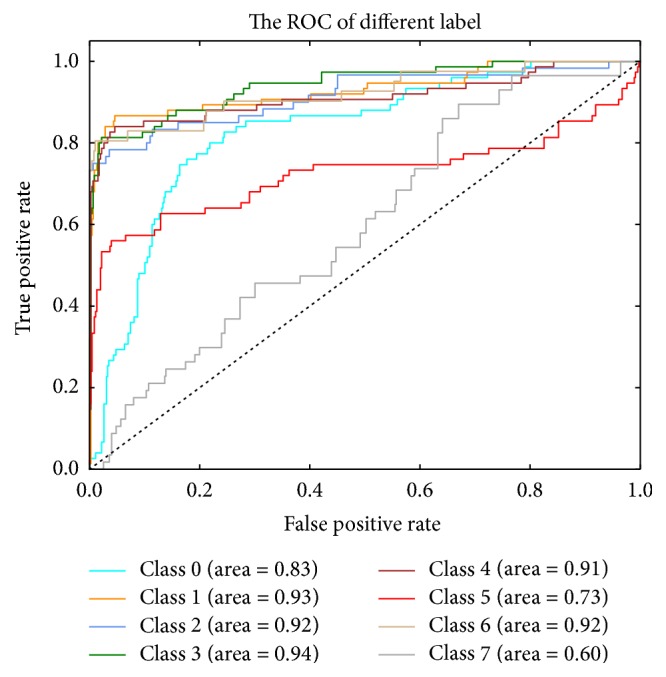
The ROC curve of each label in the Softmax based on the convolution neural network and the color feature fusion. The dotted black line is the baseline in ROC curve. It indicates that the true positive rate (TPR) is equal to the false positive rate (FPR).

The ROC curves are typically used in binary classification to study the output of a classifier. The top left corner of the plot is the “ideal” point—a false positive rate of zero and a true positive rate of one. This is not very realistic, but it does mean that a larger area under the curve (AUC) is usually better. The ROC curve of different classifiers based on the feature of convolution neural network and color feature fusion is in Figure 3. The precision-recall curve of different classifiers based on the feature of convolution neural network and color feature fusion is in Figure 4. Another evaluation measure for multiclass classification is macro-averaging and micro-averaging, which gives equal weight to the classification of each label, as shown in Figure 5. The ROC curve of each label in the Softmax classification algorithm based on the convolution neural network and the color feature fusion is shown in Figure 6.

We have done the data increment experiment on the existing dataset in this paper. In each dataset, we select 90% of the data as a training set and the remaining 10% as a test set. Under the premise of the feature of convolutional neural network and color feature fusion, the accuracy rate is gradually increasing in the same classifier with the increase of data. The experimental results are shown in Table 7.

Since the experiment of our method is based on the standard dataset whose labels were judged by the experts and the accuracy in the eight categories is 65% which is far greater than the random assignment, it indicates that there is a consistency between the experts. Secondly, the experimental process and accuracy results were reported to the experts. They believe that the proposed method is useful for practical applications. Finally, we organized a small number of volunteers to compare the body constitution recognition result of our method and that of experts, and the consistence almost keeps the same level, showing that the judgment between the different medical experts can be consistent. However, due to the small size of volunteers, the results may be influenced by random so that the large-scale contrast testing between system and experts will be expected in the future.

## 4. Conclusions

This paper presented a constitution classification algorithm based on convolutional neural networks. Our approach uses convolutional neural networks to extract the features of face images. We have also presented a set of experiments aiming to validate our algorithm. First of all, the feature extraction method of convolution neural network is better than the color and texture features. Then, under the premise of convolution neural network feature and color feature fusion, the classification of Softmax is the best by comparing different classifiers. At last, the results show that our method obtained the best results with a precision of 65.29%. As the results of the body constitution identification by CCMQ are easily influenced by the subjective factors of patients, our approach can classify body constitution faster and more accurately.

The study has shown that convolutional neural networks are effective in dealing with constitution classification based on face image. In addition, the study will serve as a reference for establishing diagnostic criteria and a diagnostic model for constitution classification and a better guide for clinical practice.

## Figures and Tables

**Table 1:** The number of samples of different constitution types.

	Gentleness	Qi-deficiency	Yang-deficiency	Yin-deficiency	Phlegm-dampness	Dampness-heat	Blood-stasis	Qi-depression	Sum
Number	570	750	600	750	750	750	410	750	5330

**Table 2:** The classification results under different feature extraction methods.

	SVM	Random Forest	KNN	Softmax	Decision Tree	Gradient BoostTree	Naive Bayes
Color feature	23.26%	25.89%	26.08%	19.14%	14.63%	19.32%	16.14%
Color and texture features	29.64%	40.87%	29.46%	22.68%	19.14%	22.89%	17.63%
CNN	63.55%	64.23%	63.23%	64.54%	60.97%	62.78%	63.78%

**Table 3:** The confusion matrix of random forest classification based on color texture feature Fusion.

	Qi-deficiency	Yin-deficiency	Yang-deficiency	Phlegm-dampness	Dampness-heat	Qi-depression	Blood-stasis	Gentleness
Qi-deficiency	38	6	6	2	7	14	0	2
Yin-deficiency	9	44	1	4	6	10	0	1
Yang-deficiency	17	10	17	2	3	8	0	3
Phlegm-dampness	17	13	1	34	4	6	0	0
Dampness-heat	10	5	2	3	45	9	0	1
Qi-depression	4	5	2	4	11	31	0	18
Blood-stasis	14	8	1	3	4	8	3	0
Gentleness	12	10	2	5	7	16	2	3

**Table 4:** The confusion matrix of Softmax classification based on convolutional neural network.

	Qi-deficiency	Yin-deficiency	Yang-deficiency	Phlegm-dampness	Dampness-heat	Qi-depression	Blood-stasis	Gentleness
Qi-deficiency	51	11	2	5	5	0	1	0
Yin-deficiency	7	61	2	2	0	2	1	0
Yang-deficiency	10	3	43	1	1	1	1	0
Phlegm-dampness	5	6	1	62	1	0	0	0
Dampness-heat	8	3	2	4	57	0	1	0
Qi-depression	1	4	0	8	3	39	0	20
Blood-stasis	5	0	0	4	2	0	30	0
Gentleness	26	10	3	6	2	7	2	1

**Table 5:** The classification results based on the convolution neural network feature extraction and color feature fusion.

	SVM	Random Forest	KNN	Softmax	Decision Tree	Gradient BoostTree	Naive Bayes
CNN	63.55%	64.23%	63.23%	64.54%	60.97%	62.78%	63.78%
CNN + color	63.98%	64.91%	62.34%	65.29%	59.85%	64.72%	63.04%

**Table 6:** The confusion matrix of Softmax classification based on convolutional neural network and color feature fusion.

	Qi-deficiency	Yin-deficiency	Yang-deficiency	Phlegm-dampness	Dampness-heat	Qi-depression	Blood-stasis	Gentleness
Qi-deficiency	36	9	2	8	12	3	5	0
Yin-deficiency	2	65	2	3	2	0	1	0
Yang-deficiency	8	2	45	1	2	1	1	0
Phlegm-dampness	4	4	0	61	3	1	2	0
Dampness-heat	7	1	1	3	63	0	0	0
Qi-depression	0	3	0	5	6	40	1	20
Blood-stasis	2	1	0	2	2	1	33	0
Gentleness	20	8	2	6	7	7	2	5

**Table 7:** The classification results with the increase of data.

	SVM	Random Forest	KNN	Naive Bayes	Softmax	Decision Tree	Gradient BoostTree
3010	44.19%	46.18%	46.84%	38.21%	43.19%	37.21%	41.86%
4470	54.36%	53.69%	52.35%	52.57%	54.14%	46.53%	53.69%
5330	63.98%	64.91%	62.34%	63.07%	65.29%	59.89%	64.72%
